# Metadata-Driven Project-Level Analysis and Noise Modeling for Amplicon Sequencing Data Using CRISPResso2

**DOI:** 10.3390/ijms27219810

**Published:** 2026-11-01

**Authors:** Olga Volodina, Svetlana Smirnikhina

**Affiliations:** Research Centre for Medical Genetics, Moscow 115522, Russia

**Keywords:** prime editing, gene editing, CRISPResso2, deep targeted sequencing

## Abstract

Here we present a lightweight workflow designed to improve the robustness and scalability of high-throughput amplicon sequencing analysis while leveraging CRISPResso2. To handle large batch processing without sensitive runtime interruptions, our pipeline processes samples sequentially, allowing the analysis to resume from the last successfully completed sample if an error occurs. The workflow implements a fallback mechanism for failed runs, automated extraction and separate processing of control samples, and a background noise model derived from control libraries within defined editing windows. Validation using spike-in datasets across Cas9, base editing, and prime editing demonstrated that our noise-adjusted estimates deviated from the ground truth by only 0.1–0.6%, whereas native CRISPResso2 caused a 1.5–3.7% overestimation. Unlike existing tools such as CRISPECTOR, which focus primarily on CRISPR-Cas9, our approach supports multiple editing modalities and enables project-level organization of large datasets. Input complexity is reduced through configurable flags and modular metadata tables, minimizing manual parameter entry. Together, these features provide a flexible, fault-tolerant, and highly accurate framework for analyzing large genome editing datasets.

## 1. Introduction

Over the past decade, genome editing technologies have undergone rapid development, fundamentally transforming both basic research and translational biomedical applications. The introduction of the CRISPR-Cas9 system represented a major breakthrough by enabling efficient, programmable, and cost-effective genome modification across a wide range of organisms [1]. Subsequent efforts have focused on improving the precision, safety, and predictability of CRISPR-Cas9-based editing, addressing limitations associated with double-strand break (DSB)-induced DNA damage and error-prone repair.

Among the most advanced next-generation genome editing approaches are base editing and prime editing, both of which aim to minimize undesired genomic alterations while enabling precise sequence changes. Base editors combine a catalytically impaired Cas protein (nickase or dead Cas9) with a DNA deaminase, allowing direct conversion of specific base pairs without introducing DSBs. Cytosine base editors (CBEs) mediate C·G to T·A transitions, whereas adenine base editors (ABEs) enable A·T to G·C conversions through deamination of adenine to inosine [2]. Although highly efficient, base editing is constrained by a limited scope of editable substitutions and may introduce bystander edits within the editing window.

Prime editing further extends the versatility of CRISPR-Cas9-based technologies by enabling targeted insertions, deletions, and all twelve possible base substitutions without relying on DSBs or donor DNA templates. This system employs a Cas9 nickase fused to a reverse transcriptase and a prime editing guide RNA (pegRNA), which encodes both target specificity and the desired edit. Following nicking of the PAM-containing (non-target) strand, the reverse transcriptase synthesizes the edited DNA sequence directly at the target locus, which is subsequently incorporated through cellular DNA repair mechanisms [3]. Prime editing is generally considered one of the safest genome editing strategies to date; however, its efficiency and fidelity remain highly dependent on experimental design and cellular context.

Following genome editing experiments, accurate assessment of editing outcomes is essential. Several experimental approaches can be employed, including measurement of target gene expression or allele-specific PCR assays. Nevertheless, sequencing-based methods remain the most precise and informative means of quantifying editing efficiency and characterizing the spectrum of on-target and unintended editing events. Both Sanger sequencing and next-generation sequencing (NGS) are commonly used for this purpose. For Sanger sequencing data, computational tools such as TIDE and TIDER enable decomposition of mixed chromatograms to estimate indel frequencies and template-directed edits, respectively [4,5].

NGS-based amplicon sequencing provides substantially higher sensitivity and resolution, particularly for low-frequency editing events. Several widely adopted bioinformatic platforms have been developed to analyze targeted CRISPR-Cas9 editing outcomes from FASTQ files. CRISPResso2 performs read quality filtering, alignment to a reference amplicon, quantification of insertions and deletions arising from non-homologous end joining (NHEJ) or homology-directed repair (HDR), frameshift analysis, and generation of summary visualizations [6]. The framework supports batch processing and comparison modes, enabling parallel analysis of multiple samples and experimental conditions. In addition, control samples can be incorporated to estimate background noise; however, normalization to controls and downstream statistical interpretation remain largely manual and experiment-specific. The developers of CRISPResso2 have also introduced CRISPRLungo, a separate pipeline for long-read amplicon sequencing data [7], which incorporates unique molecular identifier (UMI)-based error correction and statistical filtering to distinguish true editing events from background noise.

Additional tools such as CRISPECTOR and ampliCan aim to improve discrimination between true editing events and background noise arising from sequencing or PCR artifacts. CRISPECTOR implements a statistical framework that models background indel frequencies using control samples and performs hypothesis testing to identify significant editing signals [8]. While such approaches improve robustness in certain experimental contexts, most available pipelines remain primarily optimized for conventional CRISPR-Cas9 experiments and may require substantial manual configuration for other editing modalities.

In addition to CRISPResso2, several other tools have been developed for the analysis of amplicon sequencing data. ampliCan performs nuclease-optimized alignment, filters experimental artifacts, integrates controls automatically, and quantifies insertions, deletions, HDR, and targeted base editing [9]. BE-Analyzer provides dedicated analysis of base editing outcomes, including interactive visualization of mutation spectra, but does not support prime editing and uses an optional untreated control only to exclude background mutations, without statistical noise modeling [10]. TIDE and TIDER enable decomposition of Sanger sequencing chromatograms, offering a low-cost alternative when NGS is not available, but their sensitivity is limited compared with deep amplicon sequencing [4,5]. Moreover, a recent systematic comparison of Sanger-based tools (TIDE, ICE, DECODR, SeqScreener) demonstrated that their accuracy decreases for complex indels and knock-in sequences, highlighting the need for NGS-based approaches for precise quantification [11]. These tools have substantially advanced the field, yet each addresses a specific editing modality or experimental context, and most require manual configuration for heterogeneous datasets.

Other toolkits have been developed to address specific modalities or sequencing platforms. SuperDecode integrates modules for Sanger (DSDecodeMS), short-read (HiDecode), and long-read (LaDecode) data, supporting Cas9, base editing, and prime editing [12]. For base editing, BEstimate provides gRNA design and functional annotation of installed variants [13], while MultiEditRbatch enables high-throughput analysis of Sanger data [14]. For prime editing, DTMP-prime and OptiPrime use machine learning to predict pegRNA activity and editing outcomes [15,16]. Despite this progress, most tools remain focused on a single modality or sequencing platform, and few integrate control-based noise correction with project-level organization.

Beyond tool development, rigorous validation of editing quantification remains a challenge. Spike-in experiments, in which synthetic sequences with known mutation frequencies are mixed with real sequencing libraries, provide a controlled ground truth for benchmarking.

Despite the overall maturity and usability of current genome editingamplicon-sequencing analysis tools, several practical limitations remain when analyzing large experimental datasets. Widely used platforms such as CRISPResso2 provide comprehensive functionality for quantifying genome editing outcomes and support batch processing of multiple samples. However, large batch executions can become computationally heavy and operationally fragile. Errors occurring during individual runs, particularly in more complex editing scenarios such as prime editing, may interrupt the batch workflow or lead to incomplete results, requiring users to restart the analysis or manually reprocess affected samples. Although CRISPResso2 can skip previously completed runs (--no_rerun) and continue batch analysis when individual samples fail (--skip_failed), coordinating recovery, metadata, and downstream processing across heterogeneous projects still requires additional orchestration.

In addition, existing tools typically operate at the level of individual analyses rather than full experimental projects. Users must manually prepare detailed metadata tables specifying parameters for each sample and often need to process control libraries separately for every guide RNA. In experiments involving many samples, multiple guides, and different editing modalities, this workflow becomes increasingly time-consuming and prone to input errors.

To address these challenges, we developed an automated project-level framework that combines metadata-driven sample organization with robust sequential execution and control-based statistical noise modeling noise modeling. Instead of relying on a single uninterrupted batch run, samples are processed independently within a unified project structure, allowing the workflow to resume analysis from the last successfully processed sample in the event of failure. This design improves robustness in large-scale analyses while maintaining compatibility with standard outputs of CRISPResso2.

The framework is designed to scale to experiments containing hundreds of samples and multiple guide RNAs while minimizing manual intervention. It enables sensitive detection of low-frequency editing events through noise estimation derived from control libraries and predefined editing windows. All intermediate files generated by CRISPResso2 are retained, and additional outputs are produced, including project-level summary tables, statistical metrics, and corrected estimates of unintended editing within the target locus.

The proposed algorithm (https://github.com/olvolod/CRISPResso2-AntiNoise-Wrapper, accessed on 25 October 2026) is specifically designed for deep targeted sequencing rather than whole-genome sequencing data and therefore does not support CRISPRessoWGS workflows. By extending existing genome editing amplicon analysis pipelines with automated project-level orchestration and statistically principled noise modeling, this approach addresses key practical and analytical challenges in large-scale genome editing studies.

## 2. Results

### 2.1. Automated Processing of Heterogeneous Genome Editing Experiments

To evaluate the performance of the proposed framework, we applied the pipeline to a dataset containing CRISPR–Cas9, prime editing, and base editing experiments originating from multiple independent projects. The dataset included experimental samples targeting several genomic loci together with corresponding control samples.

Experimental structure was defined through metadata tables describing sample identities, guide RNAs, editing modalities, and project identifiers. This metadata-driven design enabled automatic expansion of analysis tasks and ensured consistent association between sequencing files, guide annotations, and analysis parameters.

The sample metadata table defined sample identifiers, guide associations, experimental group labels, editing modes, and project identifiers. Control samples were designated using a group value of zero. Guide-level sequence information, including spacer sequences, amplicon sequences, and pegRNA annotations, was provided in a separate guide annotation table.

Using this metadata representation, the pipeline automatically generated analysis tasks for all samples, detected corresponding FASTQ Files, and constructed CRISPResso2 commands for each experiment. This approach allowed heterogeneous editing experiments to be processed within a unified project-level framework without manual command construction.

To evaluate the performance of the noise-correction module across different editing modalities, we generated spike-in datasets with predefined mutation frequencies at five levels (Low, Med–Low, Med, Med–High, High) for CRISPR-Cas9, base editing (BE), and prime editing (PE). These datasets were derived from real control libraries by introducing defined mutations while preserving the original sequencing noise. For each sample, we compared three values: raw CRISPResso2 output, noise-adjusted output from our wrapper, and the known ground truth. This allowed us to directly assess both the systematic bias of native CRISPResso2 and the accuracy of the noise-adjusted estimates. In total, the spike-in benchmark included 15 samples (5 levels × 3 modalities), with full results provided in Supplementary Table S1.

### 2.2. Mode-Aware Processing and Fallback for Prime Editing

**Figure 1:**
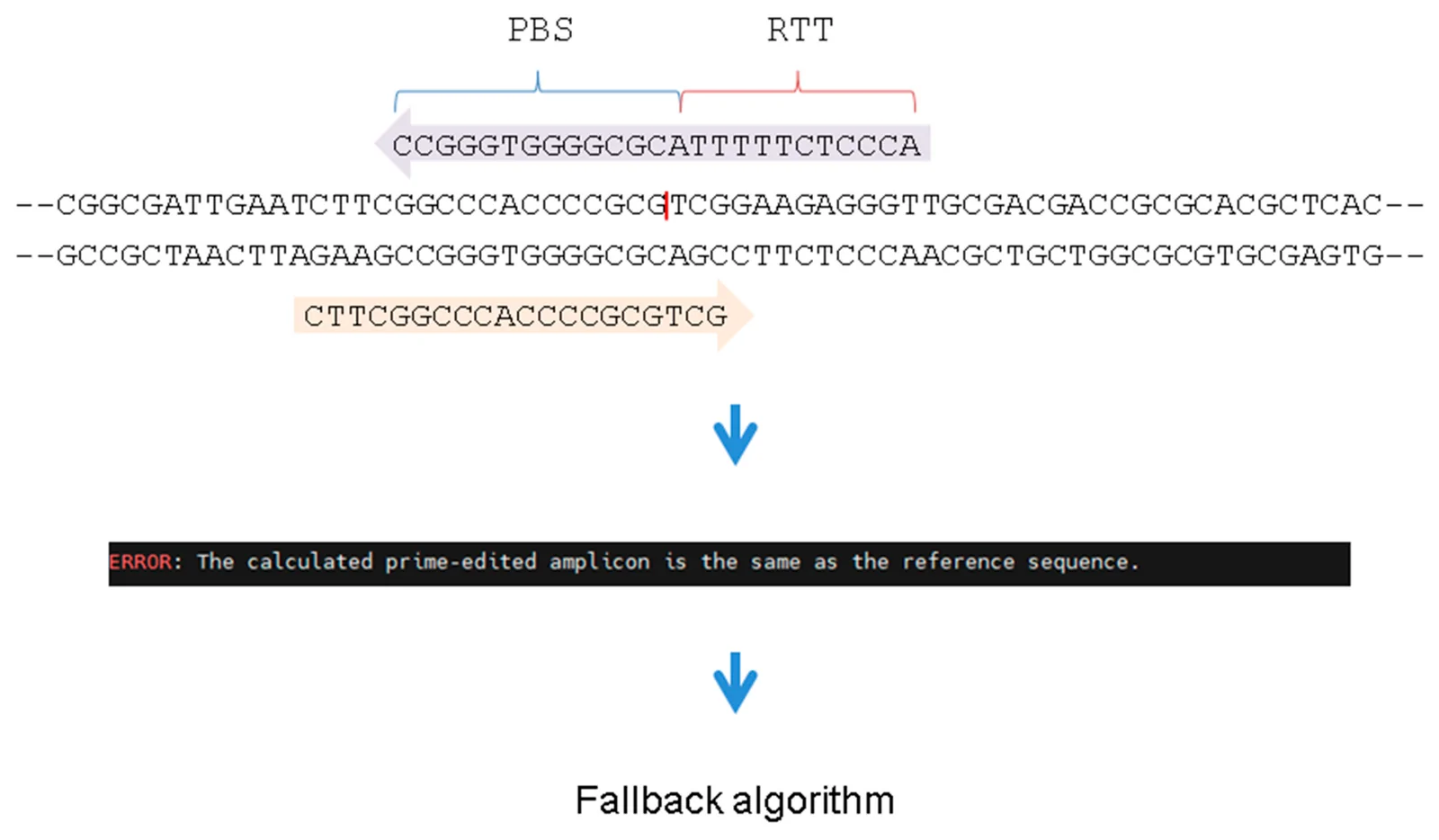
Schematic representation of the prime editing fallback strategy. Structural parts of the pegRNA: spacer (peach, with the arrow indicating the 5’-3’ direction) and extension (purple, with the arrow indicating the 5’-3’ direction) with primer binding site (PBS; blue) and reverse transcriptase template (RTT; red) regions. Native CRISPResso2 prime editing mode can fail due to alignment errors despite correctly oriented input sequences, as illustrated here. Our workflow automatically triggers a fallback to a dual-guide Cas9-based analysis, ensuring robust recovery of editing metrics.

During analysis of the prime editing dataset, CRISPResso2 prime editing mode failed for several samples because the predicted prime-edited amplicon was identical to the reference sequence according to CRISPResso2 (Figure 1). In these cases, CRISPResso2 reported that the predicted prime-edited amplicon sequence was identical to the reference sequence, preventing the pipeline from proceeding with standard analysis. To evaluate prime editing, spike-in PE samples were analyzed. Native CRISPResso2 prime editing mode failed for all PE samples due to alignment errors; consequently, data assessment was successfully performed using our custom pipeline (Table 1).

To address such failures, the framework automatically applied a fallback strategy that reprocessed affected samples using a dual-guide Cas9-based representation derived from the pegRNA structure. The first guide corresponded to the pegRNA spacer sequence, while the second represented the reverse-complemented pegRNA extension projected onto the amplicon sequence after removal of the intended edit.

Each guide was analyzed independently using CRISPResso2 in Cas9 mode, producing separate allele frequency tables corresponding to editing events in the spacer and extension regions. These allele tables were subsequently harmonized and merged to produce a unified representation of editing outcomes. Application of the fallback strategy enabled successful recovery of allele-level results for prime editing samples that failed under the native CRISPResso2 prime editing workflow.

The workflow preserved the native CRISPResso2 output directories and reports generated during both primary and fallback analyses. Following each successful run, sample-level metrics were extracted from the corresponding allele-frequency tables and stored in a unified results table. Table 1 shows representative outputs from Cas9, base-editing, and prime-editing analyses, including samples processed using the fallback configuration.

### 2.3. Control Integration and Noise Model Construction

Negative control samples (group = 0) were automatically incorporated into background estimation. Controls were associated with the corresponding project and guide during analysis. When a control sample contained a guide assignment, the control was used for that guide. When no guide was specified, the control was expanded across all guides within the same project.

Control allele-frequency tables were then aggregated to estimate background variant frequencies for each project, guide, and genomic position. The resulting noise model provides a position-specific estimate of the background frequency observed in the corresponding control samples and a statistical threshold against which experimental samples can subsequently be evaluated.

When three or more control samples were available, the control-derived background frequency was calculated from the pooled control read counts, and the threshold was defined as the upper bound of the 95% Clopper–Pearson confidence interval for the corresponding binomial proportion. When fewer than three controls were available, a Bayesian beta-binomial procedure was used with a global prior derived from the background frequency distribution across controls. The 95th percentile of the resulting posterior distribution was used as the position-specific threshold.

For each project–guide–position combination, the noise model records the estimated control-derived background frequency (control_mean), the corresponding threshold (threshold), and the number of control samples contributing to the estimate (n_controls). Projects or guide combinations for which an appropriate noise model could not be constructed were retained and reported with native CRISPResso2-derived metrics, but were not included in the noise-adjusted summary.

### 2.4. Noise-Adjusted Variant Filtering and Correction of Editing Outcomes

For each experimental sample, annotated variants were aggregated at the genomic-position level and matched to the corresponding project- and guide-specific noise model. The observed variant frequency in the sample (sample_sum) was then compared with the corresponding control-derived background frequency (control_mean) and threshold (threshold).

The over_threshold field records the result of this comparison. A value of yes indicates that the observed sample frequency exceeded the position-specific control-derived threshold, whereas no indicates that the observed frequency remained within the estimated background range. Variants that did not exceed the corresponding threshold were excluded from subsequent noise-adjusted calculations.

For alleles containing multiple positional annotations, each annotated position was evaluated independently. Only positions exceeding their corresponding background thresholds were retained. For retained positions, the lower bound of the control-derived background (the 5th posterior percentile for one or two controls or the lower limit of the two-sided 95% Clopper–Pearson interval for three or more controls) was subtracted from the observed sample frequency to obtain the noise-adjusted frequency.

Following positional filtering and background subtraction, editing metrics were recalculated. Background contributions associated with the reference sequence were quantified and subtracted from the imperfect-correction and added to the perfect-correction category.

The resulting metrics include total correction frequency, adjusted perfect-correction frequency, adjusted imperfect-correction frequency, undesired editing frequency, substitution/mutation frequency, and indel frequency. Samples belonging to projects for which no suitable noise model was available retained the corresponding native CRISPResso2-derived metrics in the results table and were not included in the noise-adjusted summary.

After noise-based filtering and background subtraction, the recalculated editing metrics were integrated into a summary table containing one row per sample with an available noise model (Table 2). Sample metadata, including project and group identifiers, were merged with the corresponding CRISPResso2-derived metrics and noise-adjusted estimates.

All values are derived from the noise-adjusted output of the wrapper (lower bound of control-derived background subtracted). HDR and prime-edited reads were not subjected to background subtraction. Full per-sample results, including all spike-in levels, are provided in Supplementary Table S1.

This unified representation allows editing outcomes from heterogeneous projects and editing modalities to be reported using a consistent sample-level structure while preserving the distinction between native CRISPResso2 results and downstream noise-adjusted results.

**Figure 2:**
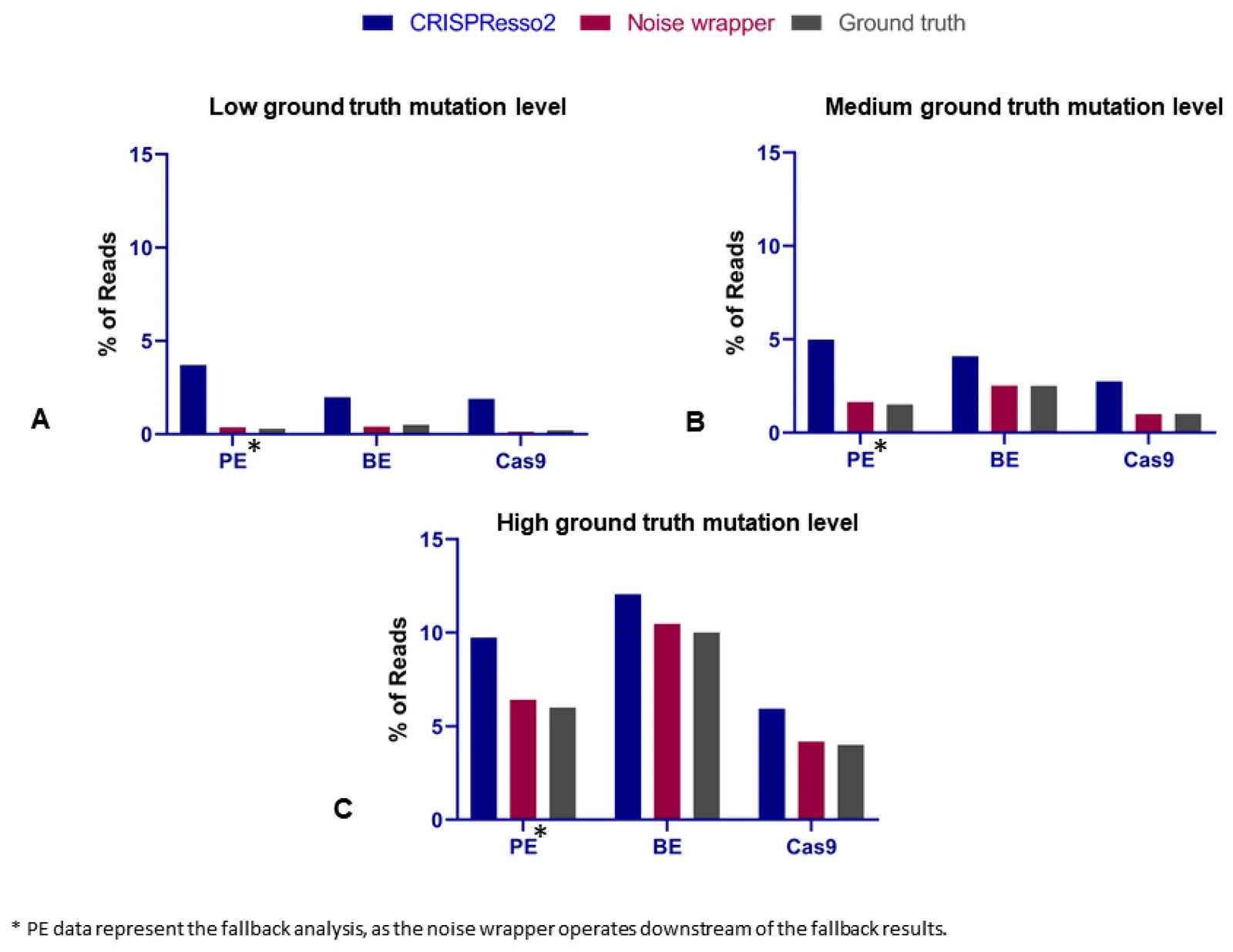
Comparison of mutation frequency estimates across CRISPR-Cas9, base editing (BE), and prime editing (PE) at low (**A**), medium (**B**), and high (**C**) spike-in mutation levels (noise model with 3 controls). Blue bars represent native CRISPResso2 results (for PE, the fallback dual-guide Cas9 analysis was used as the baseline, denoted by *). Magenta bars represent the noise-adjusted output from our wrapper. Grey bars represent the ground truth (spiked-in) mutation levels. Native CRISPResso2 systematically overestimates mutation frequencies (e.g., PE Low: ~3.7% vs. ground truth 0.3%), whereas the wrapper brings estimates into close agreement with the ground truth across all modalities and spike-in levels.

To evaluate whether the control-derived noise model improves the accuracy of mutation frequency estimates, we compared raw CRISPResso2 output, noise-adjusted output from our wrapper, and the known ground truth for the spike-in samples described above (Figure 2).

Native CRISPResso2 systematically overestimated mutation frequencies, with the discrepancy being most pronounced at low and medium spike-in levels. For example, in the PE Low sample, CRISPResso2 reported a mutation frequency of 3.71%, whereas the ground truth was 0.3%. Similarly, in the BE Low sample, CRISPResso2 reported 1.98% versus a ground truth of 0.5%. At high spike-in levels, the overestimation was smaller in relative terms but still substantial (e.g., Cas9 High: 5.94% vs. ground truth 4.0%).

In contrast, noise-adjusted estimates were within 0.1–0.6% of the ground truth across all modalities and levels (e.g., PE Low: 0.37%; BE Low: 0.40%; Cas9 High: 4.18%). This is an order of magnitude smaller than the 2–4% overestimation observed with native CRISPResso2. Notably, the wrapper did not reduce true-positive signals, as evidenced by the close agreement between adjusted estimates and ground truth at the High spike-in level.

Full per-sample results, including all spike-in levels and the underlying read counts, are provided in Supplementary Table S1.

The number of control samples had minimal effect on noise-adjusted estimates (Supplementary Figure S1). For PE, estimates decreased slightly from 1 to 2 controls (e.g., High: 6.52% to 6.40%; Med: 1.77% to 1.65%; Low: 0.50% to 0.37%) and then remained unchanged with 3 or 5 controls. For BE and Cas9, the effect was even smaller: BE High changed from 10.50% (1 control) to 10.45% (5 controls), and Cas9 High from 4.21% to 4.16%. Across all modalities, estimates stabilized at 2 controls, confirming the robustness of the Bayesian prior for small control sets.

### 2.5. Base Editing Analysis and Positional Noise Modeling

Samples processed under base editing mode were additionally evaluated at single-nucleotide resolution within the editing window. The editing window was reconstructed from CRISPResso2 output and mapped to the amplicon to determine the positions of its nucleotides. Editable positions containing the defined conversion nucleotide were identified. For each position, the frequency of all mutations, the frequency of the expected base conversion, and the fraction of the expected conversion among total mutations were calculated (Table 3).

A separate noise model was constructed for base editing controls using the same positional framework. Mutation counts, expected substitutions, and sequencing depth were aggregated per project, guide, window, and position. Noise thresholds were obtained using the statistical framework described above. For each sample, a positional matrix summarizing editing frequencies and noise thresholds was generated.

## 3. Discussion

Tools such as CRISPResso2 have become widely used for the analysis of targeted genome editing experiments, providing a framework for quantifying editing outcomes from amplicon sequencing data. CRISPResso2 supports multiple editing modalities, including Cas9-mediated editing, base editing, and prime editing, and provides batch processing for analysis of multiple samples. These features make it useful for large-scale characterization of genome editing outcomes [6]. The development of base and prime editing has further increased the need for flexible analysis workflows capable of handling different types of editing outcomes [2,3].

Large batch analyses, however, can present practical challenges. Individual samples may fail because of input inconsistencies, parameter incompatibilities, resource limitations, or runtime errors. In a large dataset, such failures can interrupt processing and require manual intervention or repeated analysis. To address these workflow-level issues, we developed a lightweight framework that extends rather than replaces CRISPResso2.

CRISPResso2 remains the underlying sequence-analysis engine responsible for read processing, alignment, editing quantification, and generation of native allele-frequency and visualization outputs. The present workflow adds a metadata-driven execution layer and downstream post-processing. These additions include sequential and resumable execution, automated fallback handling for selected prime-editing failures, automated integration of control samples, and control-derived noise adjustment.

Sequential execution was selected to improve fault isolation and resumability rather than computational speed. Each sample is processed independently, so successfully completed analyses can be retained when another sample fails. Thus, the main advantage of this design is operational robustness rather than reduced runtime.

The control-based noise model provides an additional layer of interpretation for low-frequency variants. Untransfected controls are matched to the corresponding project, guide, genomic position, and variant type, allowing the observed background to be estimated from experimentally generated sequencing data. When three or more controls are available, the upper bound of a 95% Clopper–Pearson confidence interval is used as the threshold. For one or two controls, a Bayesian model incorporates the observed counts together with a variant-type-specific prior derived from the global control distribution. The prior mean was derived from the median background frequency for the corresponding variant type, while the prior weight controlled the strength of shrinkage toward this global background estimate. This approach is conceptually related to other genome-editing analysis methods that use comparative control data and statistical modeling to distinguish editing-associated signal from experimental background [8]. However, the present implementation is designed specifically as a downstream extension of CRISPResso2-based amplicon analysis rather than as an independent off-target detection framework. In addition, the binomial model treats the aggregated control reads as observations of a common background frequency and does not explicitly model between-control heterogeneity. Therefore, the resulting thresholds represent an aggregated estimate of the background rather than a hierarchical model of control-to-control variation.

The quantitative comparison using spike-in data (Figure 2) directly demonstrates the effect of this noise-processing step. Native CRISPResso2 systematically overestimated mutation frequencies, with the discrepancy being most pronounced at low and medium spike-in levels (e.g., PE Low: 3.71% vs. ground truth 0.3%; BE Low: 1.98% vs. 0.5%). After noise adjustment, the estimates were within 0.1–0.6% of the ground truth across all modalities and levels. The small remaining deviations for most samples correspond to approximately 60–100 mutation-supporting reads lost during CRISPResso2 filtering (~0.2% of the post-processing depth). Importantly, the correction did not simply reduce all editing categories by the same proportion; the magnitude of correction depended on the overlap between the observed variant frequencies and the control-derived background.

Control-based statistical approaches have also been used in other genome-editing analysis frameworks. CRISPECTOR uses comparative treated/control NGS data and statistical modeling to estimate low-frequency editing activity and distinguish signal from experimental background [8]. It is primarily designed for CRISPR-Cas9 experiments and does not support base editing or prime editing. CRISPECTOR employs a Bayesian Maximum A Posteriori (MAP) classifier that compares treatment and mock reads at each position. The classifier models the probability that an observed indel originated from a true editing event versus background noise, using a hypergeometric distribution for the no-edit model and a binomial distribution for the edit model [8]. This approach allows CRISPECTOR to distinguish genuine low-frequency edits from sequencing artifacts. In a human-validated benchmark of 19 discordant sites, CRISPECTOR reduced false-positive and false-negative rates compared with CRISPResso2 and ampliCan, particularly at low editing frequencies (near the 0.1% activity threshold). However, this classifier is optimized for CRISPR-Cas9 indel calling and does not address substitution-based noise or support base editing and prime editing outputs. SuperDecode integrates modules for Sanger, short-read, and long-read sequencing and supports Cas9, base editing, and prime editing, but it does not implement project-level control matching or Bayesian noise modeling for low-frequency variant detection [12]. CRISPRLungo focuses on long-read sequencing data and incorporates statistical filtering when control samples are provided, but it is not designed for short-read amplicon analysis or multi-modal project-level organization [7]. In contrast, the present workflow focuses on project- and guide-matched controls within a CRISPResso2-based amplicon analysis and additionally integrates the resulting background estimates into editing summaries. The workflow therefore complements existing sequence-analysis approaches rather than attempting to replace them.

### Limitations

Several limitations should be considered. First, the metadata-driven design makes the workflow dependent on accurate sample, guide, project, and editing-mode annotations. Errors in metadata may result in incorrect associations between sequencing data, sequence parameters, and control samples.

Second, the reliability of the noise model depends on the number and quality of available controls. Larger control datasets provide more information about the background distribution, whereas estimates based on one or two controls are more dependent on the Bayesian prior. The Bayesian prior is robust to the choice of prior_weight for high-depth datasets, as the observed counts dominate the posterior. However, for very low-depth samples (e.g., <100 reads), the prior weight may have a more pronounced effect, and users should interpret such estimates with caution. The sensitivity analysis of the control sample number is presented in Supplementary Figure S1 and Table S1, and the sensitivity analysis of the Bayesian prior weight is presented only in Supplementary Table S1.

Third, the noise model can only characterize background represented by the available controls. If a variant is present in both experimental and untransfected samples, the model cannot determine whether it represents a biological event or a technical artifact. Conversely, controls that do not adequately represent the sequencing or amplification background of experimental samples may lead to incomplete background correction. The resulting noise-adjusted values should therefore be interpreted as control-informed estimates rather than independent measurements of true editing activity.

Fourth, the prime-editing fallback strategy has several limitations. (1) Alignment failure: if the reverse-complemented extension sequence cannot be unambiguously projected onto the amplicon (e.g., due to repetitive sequences, complex secondary structures, or orientation mismatch with the expected edit), the fallback cannot generate reliable results. (2) Quantification window length: if the extension quantification window is short (≤4 nt), the window may map to multiple locations in the amplicon, leading to misannotation. To address this, we implemented an automatic realignment procedure that searches for alternative positions where all modified reads have variants. Users are advised to use extension quantification windows of at least 6 nt when possible. (3) Scaffold incorporation: the fallback does not assess scaffold incorporation or other complex pegRNA-derived outcomes; it only evaluates the extra window at the end of the RTT. (4) Noise model dependency: the noise model operates on the fallback output; native CRISPResso2 prime-editing output is not processed through the noise model. In standard mode, native PE output is retained (except plots), but noise correction is not applied to native PE output. (5) Extension requirement: the fallback requires a non-empty extension sequence. If only a spacer is available, the Cas9 mode should be used instead. (6) Visualization: the fallback does not produce the native CRISPResso2 PE plots. Users are advised to inspect per-sample annotation files and to verify that the extension sequence matches the actual pegRNA design.

Fifth, spike-in mutations were introduced before CRISPResso2 processing, whereas reported frequencies are calculated on reads that passed CRISPResso2 filtering. Since CRISPResso2 discards a fraction of reads (~6.5% in our datasets), the denominator decreases and the apparent mutation frequency may be slightly inflated. This effect is most pronounced at high spike-in levels. The resulting deviations (0.1–0.6%) are smaller than the 1.5–3.7% overestimation observed with native CRISPResso2.

Sixth, HDR and prime-edited reads were not subjected to noise subtraction, as HDR events are rare and subtracting background from them could remove true biological signal. This conservative choice ensures that HDR-derived metrics are not artificially reduced.

Finally, the workflow remains dependent on the capabilities and limitations of CRISPResso2. The fallback procedure can recover selected prime-editing analyses that fail under the native configuration, which was one of the motivations for developing the workflow, but successful recovery cannot be guaranteed for all experimental designs or parameter combinations.

Taken together, the workflow extends CRISPResso2-based analysis with metadata-driven execution, failure recovery, automated control integration, and control-derived noise adjustment. The results demonstrate that this additional processing can substantially change the estimated frequency of low-level undesired variants in datasets with measurable background signal. The main contribution of the workflow is therefore not a replacement for CRISPResso2, but an additional layer for fault-tolerant processing and control-informed interpretation of heterogeneous amplicon-sequencing datasets.

## 4. Materials and Methods

### 4.1. Pipeline Overview

We developed a project-level orchestration and post-processing framework built on top of CRISPResso2 for automated analysis of targeted amplicon sequencing data generated in CRISPR-Cas9, base editing, and prime editing experiments. The system integrates experimental metadata, guide annotations, and parameter configurations to enable scalable batch processing while preserving all native CRISPResso2 outputs.

The workflow automatically detects FASTQ files, expands samples into analysis tasks based on metadata, generates mode-specific CRISPResso2 commands, executes analyses, and performs standardized post-processing of allele frequency tables. Results are saved as each sample is processed, allowing completed analyses to be retained if a later sample fails. The pipeline was initially validated on three experimental projects (9 prime-editing, 7 CRISPR-Cas9, and 4 base-editing samples) to confirm its ability to process heterogeneous datasets. Quantitative benchmarking of noise correction was performed separately using spike-in datasets (see below and Supplementary Table S1).

### 4.2. Input Data and Task Expansion

The sample metadata table defines the sample-level information used to organize the analysis and to associate each sequencing sample with the corresponding experimental conditions. Each row represents one sequencing sample and contains a unique sample identifier (sample_name), the associated guide RNA (guide_id), the experimental group (group), the editing modality (edit_mode), and the project identifier (project).

The group field is used to separate experimental samples from negative controls. A value of group = 0 means a negative control sample, whereas non-zero group values identify experimental groups. The edit_mode field specifies the editing modality analyzed by CRISPResso2 and by the corresponding downstream workflow components, including CRISPR-Cas9, base editing, and prime editing.

Guide assignment is optional for negative control samples. When a negative control is not associated with a specific guide RNA, the workflow automatically associates that control with all guides defined within the same project. This allows a single project-level control to be used for estimating background variation across multiple guide-specific analyses without requiring duplicate metadata entries.

The project field defines the analysis context in which samples, controls, guide annotations, and amplicon sequences are grouped. This project-level organization is central to the metadata-driven design of the workflow because it allows the pipeline to process heterogeneous datasets while maintaining the relationships between samples, guides, editing modalities, and corresponding controls (Supplementary Table S2).

The guide and sequence annotation table contains the sequence-level information required to construct the CRISPResso2 analysis for each guide and project. The table separates guide-specific information from project-specific sequence information while linking the two through the corresponding identifiers.

The guide_id field uniquely identifies a guide RNA and is associated with its spacer sequence. For prime-editing experiments, the optional scaffold and extension fields contain the corresponding pegRNA-associated sequences. These fields are left empty when they are not applicable to the selected editing modality.

The project_id field identifies the project to which the corresponding amplicon belongs. The amplicon field contains the reference amplicon sequence used for the analysis, while expected_seq contains the expected edited sequence when such a sequence is required for the selected analysis. The expected_seq field may therefore remain empty for projects where an expected edited sequence is not defined or is not required.

This organization allows guide-specific information to be reused across samples associated with the same guide, while project-specific amplicon and expected-sequence information is associated with the corresponding project. The workflow uses these identifiers to retrieve the appropriate sequences when constructing CRISPResso2 commands and to avoid requiring the same sequence information to be duplicated across individual sample records.

All nucleotide sequence inputs are restricted to the characters A, T, G, and C (case-insensitive); ambiguous nucleotide symbols are not accepted. Guide and project identifiers are unique numeric identifiers within the metadata structure (Supplementary Table S3).

A separate parameter table defines the CRISPResso2 configuration using a key-value structure. The table separates parameters that are shared across the analysis from project-specific parameter overrides. The parameters column contains the CRISPResso2 command-line parameter, while the values column specifies its default value. Unless an override is specified, the value in this column is applied uniformly across projects.

The project column identifies projects for which the default parameter value is overridden. The change column specifies the alternative value used for the corresponding project or projects. Thus, the table allows a common set of CRISPResso2 parameters to be defined once while permitting project-specific modifications when experimental requirements differ.

For example, --default_min_aln_score has a default value of 60, whereas project 3 uses an alternative value of 50. Similarly, project-specific values can be defined for parameters such as -q or other CRISPResso2 options when required (Supplementary Table S4).

Some parameters are marked as additional in the values column. These parameters are not part of the common default configuration and are instead enabled only for the projects specified in the subsequent project-specific columns. This mechanism allows optional CRISPResso2 functionality to be activated selectively without affecting projects for which the parameter is not applicable.

During command construction, the workflow combines the default parameter set with any project-specific overrides. Parameters that are incompatible with the selected editing modality are excluded before execution. This structure allows the same metadata-driven workflow to accommodate heterogeneous projects while keeping the core analysis configuration standardized and project-specific deviations explicit.

### 4.3. Execution Modes and Fallback Strategy

The pipeline supports two execution modes: standard and noise-corrected. The execution mode is determined on a per-sample basis via the noise column (containing yes or no) in the samples.csv metadata table.

In standard mode, samples are processed using project-specific CRISPResso2 configurations. For prime editing datasets, an automated fallback strategy is triggered if CRISPResso2 fails due to alignment errors or complex sequence structures. The fallback performs a dual-guide analysis in Cas9 mode using two guide definitions derived from the pegRNA structure: the spacer sequence and a reverse-complemented extension sequence projected onto the amplicon after removal of the intended edit. For the extension guide, the quantification window is forced to start at the end of the RTT (-wc 0), mimicking the behavior of the native CRISPResso2 prime editing mode. This ensures that the window captures the region where prime editing is expected to occur, rather than the spacer-proximal region. Each run generates an independent allele frequency table.

The fallback serves two purposes. In standard mode, it ensures recovery of allele tables when prime analysis fails. In noise-corrected workflows, it provides a consistent dual-guide representation that is directly used for noise modeling and statistical filtering.

In noise-corrected mode, all samples are processed using a unified Cas9-based strategy. Prime samples are analyzed through the same dual-guide approach, and background mutation frequencies are estimated from control samples. Technology-specific CRISPResso2 configurations are not used. Instead, a standardized analysis framework ensures comparability across editing modalities.

### 4.4. Spike-In Benchmark Design

To evaluate the accuracy of noise correction, we generated spike-in datasets with predefined mutation frequencies across five levels (Low, Med–Low, Med, Med–High, High) for each editing modality (CRISPR-Cas9, base editing, and prime editing). For each level and modality, a set of control libraries from a real experiment was used as the background. The original sequences were masked by nucleotide substitutions to avoid any resemblance to the real genomic loci, and predefined mutations, insertions, deletions, and corrections were introduced at the desired frequencies. The original sequencing noise (quality scores, read length distribution, and depth) was preserved.

The total number of read pairs in each dataset was 61,617. At the Med level, Cas9 contained 1.5% perfect correction, 0.5% imperfect mutation, 1.5% imperfect insertion, 1.5% imperfect deletion, 2.5% background deletion, 2.5% background insertion, and 1.0% background mutation (“background” here refers to undesired non-correction events introduced in the ground truth rather than control noise). PE contained 2.5% perfect correction, 1.5% imperfect mutation, 0.5% imperfect insertion, 0.5% imperfect deletion, 0.15% background deletion, 0.05% background insertion, and 1.5% background mutation. BE contained 2.5% perfect correction, 2.5% imperfect mutation, and 2.5% background mutation, with no indels. Other levels (Low, Med–Low, Med–High, and High) were scaled proportionally for PE and BE. For Cas9 High, the predefined frequencies differed slightly from exact proportional scaling due to a limited number of available reads (Supplementary Table S1).

All spike-in datasets, including the ground-truth frequencies, raw CRISPResso2 output, and noise-adjusted estimates, are provided in Supplementary Table S1 (Excel file).

### 4.5. Control-Based Noise Modeling

Untransfected control samples (group = 0) are automatically incorporated for background estimation. Controls are processed in a guide-dependent manner: if a guide is specified, the control is analyzed using that guide; otherwise, it is expanded across all project-associated guides during CRISPResso2 analysis.

Control allele tables are parsed, and variant counts are aggregated by project, guide, genomic position, and variant type. For each combination of project, guide, and position, the number of variant-supporting reads and the total sequencing depth are pooled across the available control samples:

k = sum(control variant reads)

*n* = sum(control sequencing depth)

The observed pooled control frequency is:

p_hat = k/*n*

Noise thresholds are estimated separately for each project, guide, and genomic position. When three or more independent control samples are available, the upper bound of a 95% Clopper–Pearson binomial confidence interval is used as the noise threshold (T = Beta_inverse(0.975, k + 1, *n* − k)), where Beta_inverse denotes the inverse cumulative distribution function of the beta distribution. The corresponding control-derived background frequency is calculated as p_noise = k/*n*. When one or two control samples are available, a Bayesian model is used instead of the binomial confidence interval. A beta prior is parameterized using the median background frequency (global_p) and prior weight (prior_weight) estimated for the corresponding variant type from the global control dataset:

alpha_0 = global_p × prior_weight

beta_0 = (1-global_p) × prior_weight

The pooled control observations are then incorporated into the prior to obtain the posterior distribution:

alpha_post = alpha_0 + k

beta_post = beta_0 + (*n* − k)

The posterior mean is used as the estimated background frequency p_noise = alpha_post/(alpha_post + beta_post), and the noise threshold is defined as the 95th posterior quantile T = Beta_inverse(0.95, alpha_post, beta_post). Thus, the Bayesian procedure incorporates information from the available control observations while regularizing estimates using the global background distribution for the corresponding variant type.

For both approaches, the resulting noise model records the estimated background frequency, the corresponding threshold, and the number of contributing control samples. If no suitable control-derived model is available for a project, no noise correction is applied and the corresponding samples retain their native CRISPResso2-derived metrics.

In addition to the upper bound (used as a filtering threshold), the lower bound of the same confidence interval (5th percentile for the Bayesian model, or the lower limit of the Clopper–Pearson interval) was computed for each position. For noise-adjusted estimates, this lower bound was subtracted from the observed variant frequency, providing a conservative correction that avoids underestimation of true editing signal.

Observed variant frequencies in experimental samples are subsequently compared with the corresponding project-, guide-, and position-specific thresholds. Variants exceeding the estimated background threshold are retained for downstream analysis, whereas variants at or below the threshold are considered consistent with the control-derived background and are excluded from noise-adjusted editing estimates.

### 4.6. Variant Annotation and Fallback Processing

Allele frequency tables generated by CRISPResso2 are parsed, and only reads classified as MODIFIED are retained. Aligned reads are compared to the reference amplicon within the quantification window and annotated at nucleotide resolution.

Variants are classified as substitutions, insertions, or deletions and annotated with two attributes: a nucleotide-level change (e.g., A > C, delG, insT) and a positional identifier relative to the amplicon (e.g., mut118, del45). Multiple variant events within a read are stored as semicolon-separated annotations. Adjacent variants of the same type are collapsed into continuous positional ranges to reduce redundancy.

In the prime fallback workflow, allele tables from spacer and extension analyses are harmonized. Overlapping positions between quantification windows are identified and filtered to avoid double counting. The spacer table is treated as the primary dataset, as it contains the full set of alleles observed across all reads. The extension table is used to refine spacer annotations: if an allele is classified as MODIFIED in the extension table but appears as UNMODIFIED in the spacer table, its status in the spacer table is updated to MODIFIED, and the corresponding n_deleted, n_inserted, and n_mutated counts are transferred from the extension table. Alleles present only in the extension table (not observed in the spacer table) are not merged into the spacer dataset; instead, they are saved separately as extension-only alleles for transparency. After refinement, the updated spacer table is used for downstream quantification.

#### Software Availability

The CRISPResso2 AntiNoise Wrapper is available at https://github.com/olvolod/CRISPResso2-AntiNoise-Wrapper (accessed on 25 October 2026). It runs on Linux with Python 3.10+ and requires Bioconda, CRISPResso2 2.2.12+, Biopython, and Statsmodels. There are no restrictions on use by non-academics.

### 4.7. Noise Filtering and Editing Quantification

For experimental samples, position-specific variant frequencies are compared with control-derived noise thresholds. Variants below the threshold are removed. If multi-position variant annotations contain both significant and insignificant positions, only positions exceeding the threshold are retained.

Background-adjusted frequencies were computed by subtracting the lower bound of the control-derived noise (the 5th posterior percentile for one or two controls or the lower limit of the two-sided 95% Clopper–Pearson interval for three or more controls) from the observed sample frequency. HDR and prime-edited reads were not adjusted.

HDR outcomes were retained from the native CRISPResso2 output without noise subtraction. For reference-derived alleles, background mutation events were quantified and reassigned from imperfect correction to perfect correction estimates.

Final metrics include total correction frequency, perfect and imperfect correction frequencies, undesired editing frequency, and aggregated substitution, insertion, and deletion frequencies.

### 4.8. Final Result Integration

All calculated metrics were merged with experimental metadata into a unified project-level summary table containing one row per sample. Metadata included project identifier, guide RNA, and experimental group. Samples from projects without available controls were reported using raw CRISPResso2 metrics without noise correction. Control samples were reported separately and did not receive adjusted editing estimates. All intermediate files, including allele tables, annotation outputs, noise models, and positional matrices, were retained to ensure reproducibility and traceability of analysis.

### 4.9. Base Editing Positional Analysis

For samples analyzed in base editing mode, editing outcomes are evaluated at single-nucleotide resolution within the editing window. The window sequence reported by CRISPResso2 is mapped onto the amplicon to determine the positions of its nucleotides.

Editable positions containing the conversion nucleotide defined by the base editor (e.g., G for G > C editing) are identified. For each position, mutation statistics are computed including the frequency of all mutations, the frequency of the expected base conversion, and the fraction of expected substitutions among total mutations.

An additional noise model is constructed from control samples of base editing projects by aggregating mutation counts, expected substitutions, and sequencing depth per project, guide, window, and position. For each sample, a positional matrix summarizing mutation frequencies, control frequencies, noise thresholds, and an over-threshold indicator is generated and exported for downstream analysis. Tables are saved separately.

### 4.10. Sensitivity Analysis

Sensitivity analysis of the prior_weight parameter was performed for samples analyzed with a single control sample (i.e., where the Bayesian beta-binomial model is applied). The prior_weight was varied across 1, 10, 50, and 100 (Supplementary Table S1).

### 4.11. Gene-Editing Materials

Deep targeted sequencing data were generated from genome editing experiments performed on patient-derived cells. Sequencing libraries were subjected to targeted amplicon sequencing for downstream computational analysis.

## 5. Conclusions

We developed a metadata-driven computational workflow for automated and fault-tolerant analysis of heterogeneous genome editing amplicon-sequencing datasets, including CRISPR-Cas9, base-editing, and prime-editing experiments. Built upon CRISPResso2, our workflow introduces sequential and resumable execution, automated fallbacks for specific prime-editing errors, and integrated control-derived noise adjustment.

The pipeline was validated using spike-in datasets with predefined mutation frequencies across five levels (Low to High) for all three editing modalities. Native CRISPResso2 systematically overestimated mutation frequencies (e.g., PE Low: 3.71% vs. ground truth 0.3%), whereas noise-adjusted estimates deviated from the ground truth by only 0.1–0.6%. This demonstrates that the control-derived post-processing can substantially reduce background-associated mutation and undesired-editing signals while preserving the spiked-in signal. Sensitivity analysis showed that, in the tested datasets, estimates stabilize with as few as two control samples, confirming the robustness of the Bayesian prior for small control sets.

## Figures and Tables

**Table 1:** Example of CRISPResso2 execution and fallback behavior.

Sample	Correction_%	Perfect_Correction_%	Imperfect_Correction_%	Undesired_%	Indel_%	Mutation_%	Reads	QuantWindow
spike_CRISPR_high	20.14	6.37	13.78	16.57	10.64	5.94	57,618	CCCGCGTCGGAA
spike_CRISPR_low	1.05	0.31	0.74	2.99	1.1	1.89	57,618	CCCGCGTCGGAA
spike_CRISPR_med	5.3	1.58	3.72	8.06	5.32	2.74	57,617	CCCGCGTCGGAA
spike_base_high	21.25	10.62	10.63	12.08	0.03	12.05	57,616	CCCGCGTCGGAA
spike_base_low	1.1	0.57	0.53	2.01	0.03	1.98	57,616	CCCGCGTCGGAA
spike_base_med	5.35	2.69	2.66	4.13	0.03	4.1	57,616	CCCGCGTCGGAA
spike_prime_high_fallback	21.21	10.58	10.63	10.71	0.98	9.73	57,616	CCCGCGTCGGAAGGGTTGCG
spike_prime_low_fallback	1.05	0.53	0.52	3.87	0.17	3.71	57,616	CCCGCGTCGGAAGGGTTGCG
spike_prime_med_fallback	5.3	2.64	2.66	5.32	0.34	4.98	57,617	CCCGCGTCGGAAGGGTTGCG

sample, sample identifier, including the fallback designation where applicable; correction_%, total correction frequency calculated from the corresponding allele-frequency results; perfect_correction_%, frequency of alleles classified as perfect correction; imperfect_correction_%, frequency of alleles classified as imperfect correction; undesired_%, frequency of undesired editing outcomes; Indel_%, frequency of insertion/deletion outcomes; Mutation_%, frequency of substitution/mutation outcomes; reads, number of reads included in the corresponding analysis; QuantWindow, sequence or positional definition of the quantification window used for the analysis. Values shown in this table are derived from native CRISPResso2 output (for prime editing, from the fallback dual-guide analysis) and are not noise-adjusted values.

**Table 2:** Noise-adjusted editing summary.

Sample	Correction	p_Correction_%	i_Correction_%	Undesired_%	Indel_%	Mutation_%	Guide	Group
spike_CRISPR_high	20.14	6.36	13.78	14.78	10.6	4.18	3	1
spike_CRISPR_low	1.05	0.31	0.74	1.2	1.07	0.13	3	1
spike_CRISPR_med	5.3	1.58	3.72	6.26	5.28	0.99	3	1
spike_base_high	21.25	10.62	10.63	10.47	0	10.47	1	1
spike_base_low	1.1	0.57	0.53	0.4	0	0.4	1	1
spike_base_med	5.35	2.69	2.66	2.52	0	2.52	1	1
spike_prime_high	21.21	10.58	10.63	7.25	0.86	6.4	2	1
spike_prime_low	1.05	0.53	0.52	0.42	0.05	0.37	2	1
spike_prime_med	5.3	2.64	2.66	1.86	0.22	1.65	2	1

sample, sample identifier; correction, total correction frequency (perfect + imperfect) after noise adjustment, %; p_correction_%, frequency of perfect correction after noise adjustment, %; i_correction_%, frequency of imperfect correction after noise adjustment, %; undesired_%, frequency of undesired editing outcomes (indels + substitutions outside the expected edit) after noise adjustment, %; indel_%, frequency of insertions/deletions after noise adjustment, %; mutation_%, frequency of substitutions after noise adjustment, %; guide, guide RNA identifier; group, experimental group identifier (1 = experimental sample).

**Table 3:** Base editing additional results.

Window	C	C	C	G	C	G	T	C	G	G	A	A
pos	66	67	68	69	70	71	72	73	74	75	76	77
percent_sample	N/A	N/A	N/A	0.01	N/A	0.01	N/A	N/A	0.53	0	N/A	N/A
percent_sample_pos	N/A	N/A	N/A	0.11	N/A	0.14	N/A	N/A	0.53	0.18	N/A	N/A
percent_sample_from_mut	N/A	N/A	N/A	11.48	N/A	7.59	N/A	N/A	100	0	N/A	N/A
percent_control	N/A	N/A	N/A	0.01	N/A	0.03	N/A	N/A	0.01	0	N/A	N/A
percent_control_pos	N/A	N/A	N/A	0.16	N/A	0.23	N/A	N/A	0.15	0.22	N/A	N/A
percent_control_from_mut	N/A	N/A	N/A	6.56	N/A	13.41	N/A	N/A	9.61	0	N/A	N/A
over_threshold	no	no	no	no	yes	no	no	no	yes	no	yes	no

window, nucleotide sequence defining the analyzed editing window; pos, 1-based position in the reference amplicon; percent_sample, frequency of the expected base conversion in the experimental sample at the corresponding position; percent_sample_pos, total mutation frequency at that position; percent_sample_from_mut, proportion of the expected conversion among all observed mutations; percent_control, frequency of the expected base conversion in control samples; percent_control_pos, total mutation frequency in controls; percent_control_from_mut, proportion of the expected conversion among control mutations; over_threshold, indicates whether the substitution frequency at the corresponding position in the experimental sample exceeds the control-derived noise threshold (evaluated for all positions, including non-editable ones); N/A, not applicable (position does not contain the conversion nucleotide).

## Data Availability

Data available in a publicly accessible repository.

## Supplementary Materials

The following supporting information can be downloaded at: https://www.mdpi.com/article/10.3390/ijms27219810/s1.

## Abbreviations

The following abbreviations are used in this manuscript:

CRISPR	Clustered Regularly Interspaced Short Palindromic Repeats
DSB	double-strand break
ABE	adenine base editor
CBE	cytosine base editor
PCR	polymerase chain reaction
NGS	next-generation sequencing
HDR	homology-directed repair
NHEJ	non-homologous end joining
pegRNA	prime editing guide RNA
PBS	primer binding site
RTT	reverse transcription template
BE	base editing/base editor
PE	prime editing/prime editor
UMI	unique molecular identifier
MAP	maximum a posteriori
gRNA	guide RNA
WGS	whole-genome sequencing
nt	nucleotide/nucleotides
