# Supplementary material for: Metadata-Driven Project-Level Analysis and Noise Modeling for Amplicon Sequencing Data Using CRISPResso2
Source: Int J Mol Sci. 2026 Nov 1;27(21):9810. doi: 10.3390/ijms27219810 (PMC13763569; doi:10.3390/ijms27219810)
Supplement: Supplementary file 1 [file ijms-27-09810-s001.zip › Supplementary.pdf]

Supplementary

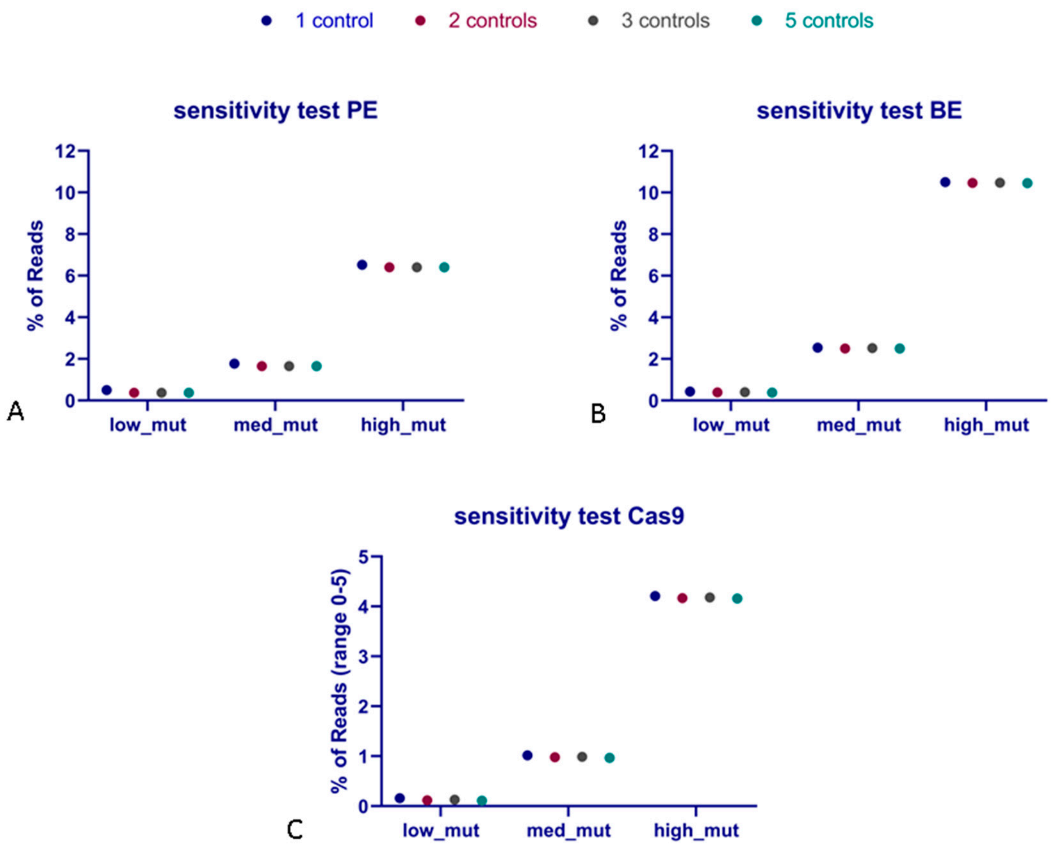

Supplementary Figure S1. Sensitivity analysis of the noise model across three editing modalities. Mutation frequency estimates are shown for 1, 2, 3, and 5 control samples at low, medium, and high spike-in levels for (A) PE, (B) BE, and (C) Cas9. Estimates are highly stable across different numbers of controls, demonstrating the robustness of the Bayesian prior for small control sets.

Table S2. Sample metadata structure used for project-level analysis. sample_name, unique sample identifier (derived from FASTQ filenames); guide_id, guide RNA identifier (referenced from Table S2; may be empty for control samples); group, experimental group identifier (0 = negative control, 1+ = experimental replicates); edit_mode, editing modality (cas9, base, or prime); project, project identifier (referenced from Table S2); noise, indicates whether the sample is processed through the noise-correction pipeline (yes or no).

sample_name	guide_id	group	edit_mode	project	noise
spike_CRISPR_high	3	1	cas9	3	yes
spike_CRISPR_low	3	1	cas9	3	yes
spike_CRISPR_med	3	1	cas9	3	yes
spike_base_high	1	1	base	1	yes
spike_base_low	1	1	base	1	yes
spike_base_med	1	1	base	1	yes
spike_prime_high	2	1	prime	2	yes
spike_prime_low	2	1	prime	2	yes
spike_prime_med	2	1	prime	2	yes

masked_control_1	0	cas9	3	yes
masked_control_1	0	base	1	yes
masked_control_1	0	prime	2	yes
masked_control_2	0	cas9	3	yes
masked_control_2	0	base	1	yes
masked_control_2	0	prime	2	yes
masked_control_3	0	cas9	3	yes
masked_control_3	0	base	1	yes
masked_control_3	0	prime	2	yes

Table S3. Guide and project-level sequence annotations used to construct CRISPResso2 analyses. guide_id, unique guide identifier; spacer, guide spacer sequence (required for all projects); scaffold, pegRNA scaffold sequence (prime editing only; leave empty otherwise); extension, pegRNA extension sequence (prime editing only; leave empty otherwise); project_id, project identifier; amplicon, reference amplicon sequence for the project; expected_seq, expected edited sequence (optional; may be empty if no expected edit is defined).

guide_id	spacer	scaffold	extension	project_id	Amplicon	expected_seq
1	spacer sequenc e 1	(should be empty, if the project does not contain pegRNA)	(should be empty, if the project does not contain pegRNA)	1	amplicon sequence for project 1	expected sequence for project 1
2	spaser sequenc e 2	scaffold sequence 2	extension seq 2	2	amplicon sequence for project 2	expected sequence for project 2
3	spaser sequenc e 3	scaffold sequence 3	extension seq 3	3	amplicon sequence for project 3	(should be empty, if the project does not contain expected sequence)
4	spacer sequenc e 4					

Table S4. Parameters table used to construct CRISPResso2 analyses. parameters, CRISPResso2 command-line parameter; values, default value applied to all projects (unless overridden); project, project identifier for which the default value is overridden; change, alternative value used for the specified project(s). If values is set to additional, the parameter is enabled only for the projects listed in project (with an optional value in change). If change is set to false, the parameter is removed for the specified project.

parameters	values	project	change
--default_min_aln_score	60		
-q	30		
-s	0		
--plot_window_size	20	2	10
--min_bp_quality_or_N	0		
--exclude_bp_from_left	15	2	5

--exclude_bp_from_right	15	2	5
-w	6		
-wc	-3		
--write_cleaned_report			
--place_report_in_output_folder			
--prime_editing_pegRNA_extension_quantification_window_size	4		
--conversion_nuc_from	G		
--conversion_nuc_to	C		
--suppress plots			
